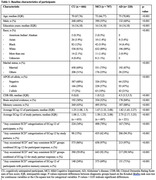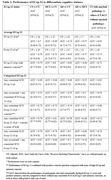# Performance of a short version of the Everyday Cognition scale (ECog‐12) to detect cognitive impairment

**DOI:** 10.1002/alz.087090

**Published:** 2025-01-03

**Authors:** Manchumad Manjavong, Adam Diaz, Joseph Eichenbaum, Miriam T. Ashford, Anna Aaronson, Melanie J. Miller, Jae Myeong Kang, Scott R Mackin, Rachana Tank, Bernard Landavazo, Diana Truran‐Sacrey, Sarah Tomaszewski Farias, Michael S. W. Weiner, Rachel L. Nosheny

**Affiliations:** ^1^ University of California, San Francisco, San Francisco, CA USA; ^2^ Khon Kaen University, Khon Kaen Thailand; ^3^ Northern California Institute for Research and Education (NCIRE), San Francisco, CA USA; ^4^ San Francisco Veterans Affairs Medical Center, San Francisco, CA USA; ^5^ Veterans Affairs Medical Center, San Francisco, CA USA; ^6^ NCIRE Northern California Institute for Research education, San Francisco, CA USA; ^7^ Gachon University Gil Medical Center, Incheon Korea, Republic of (South); ^8^ Center for Imaging of Neurodegenerative Diseases, San Francisco Veterans Affairs Medical Center, San Francisco, CA USA; ^9^ Mental Health Service, Department of Veterans Affairs Medical Center, San Francisco, CA USA; ^10^ University of California, San Francisco Department of Psychiatry, San Francisco, CA USA; ^11^ Dementia Research Center UCL Institute of Neurology University College London, London United Kingdom; ^12^ University of California, Davis, Sacramento, CA USA; ^13^ VA Advanced Imaging Research Center, San Francisco Veterans Affairs Medical Center, San Francisco, CA USA

## Abstract

**Background:**

The Everyday Cognition questionnaire is a sensitive tool for measuring decline in activities of daily living and can differentiate dementia from normal cognition. There is limited data on comparing the ECog‐12 performance by raters (self vs. informant/study‐partner) and scoring systems (continuous average score vs. a categorical grouping) to differentiate cognitive statuses.

**Method:**

ECog‐12 data were extracted from the 39‐item version collected in the Alzheimer’s Disease Neuroimaging Initiative. Participants (n = 1593) were divided into subgroups based on diagnosis. The average and categorized ECog‐12 scores were calculated. Logistic regression was used to evaluate the ECog‐12 performance in five different diagnostic groups, including discriminating between cognitively unimpaired (CU; n = 666) vs. cognitive impairment (CI; MCI and AD), CU vs. MCI (n = 707), CU vs. AD (n = 220), AD vs. MCI, and CI with amyloid pathology (n = 532) vs others. Simple logistic regression models with the ECog‐12 score as the sole independent variable were used to evaluate the association with CI, and multiple logistic regression models with participant characteristics included as covariates. This procedure was repeated for self/participants (PT) and study‐partners (SP; n = 1571) reports.

**Result:**

ECog‐12 cut‐off scores of 1.36 (PT) and 1.45 (SP) distinguish CU from CI with AUC 0.7 (0.68‐0.73) and 0.78 (0.76‐0.8). Adding a memory‐concern question to the ECog‐12 of PT increased the AUC to 0.78. Any consistent SCD (categorized scores) of PT and SP provided AUC 0.69 (0.67‐0.71) and 0.78 (0.76‐0.8). Average ECog‐12 of PT and SP can differentiate between MCI and CU (AUC: 0.69, 0.75), AD and CU (AUC: 0.74, 0.93), AD and MCI (AUC: 0.57, 0.77), and amyloid‐positive CI and others (AUC: 0.65, 0.75). Average ECog‐12 of SP had a higher association with CI than PT (OR 35.45 vs. 8.79, 95%CI 22.41‐58.03 and 6.32‐12.43, respectively).

**Conclusion:**

The ECog‐12 of SP showed higher performance in differentiating between the different cognitive statuses and higher prognostic value than that of PT. A memory concern question enhanced the performance of ECog‐12 of PT. The ECog‐12 (PT and/or SP) could be a valuable tool for quickly (and potentially remotely) assessing a person’s cognitive status for research studies and in clinical practice.